# White matter integrity moderates the relation between experienced childhood maltreatment and fathers’ behavioral response to infant crying

**DOI:** 10.1002/dev.22058

**Published:** 2020-11-17

**Authors:** Kim Alyousefi‐van Dijk, Noa van der Knaap, Renate S.M. Buisman, Lisa I. Horstman, Anna M. Lotz, Madelon M. E. Riem, Carlo Schuengel, Marinus H. van IJzendoorn, Marian J. Bakermans‐Kranenburg

**Affiliations:** ^1^ Clinical Child & Family Studies Faculty of Behavioral and Movement Sciences Vrije Universiteit Amsterdam The Netherlands; ^2^ Leiden Institute for Brain and Cognition Leiden University Medical Center Leiden The Netherlands; ^3^ Department of Psychology, Education, and Child Studies Erasmus University Rotterdam Rotterdam The Netherlands; ^4^ Behavioral Science Institute Radboud University Nijmegen The Netherlands

**Keywords:** brain imaging, early experience, parental care, perinatal

## Abstract

The ability to provide appropriate responses to infant distress is vital to paternal care, but may be affected by fathers’ experiences of childhood maltreatment. Detrimental effects of childhood maltreatment have been found in the adult brain's white matter fibers, accompanied with impaired emotional and cognitive functioning. In the current study (*N* = 121), we examined new and expectant fathers’ childhood maltreatment experiences (i.e. emotional and physical abuse and neglect), current behavioral responses (i.e. handgrip force) to infant cry sounds, and white matter integrity using diffusion tensor imaging. First, more exposure to childhood maltreatment was associated with more use of excessive handgrip force in response to infant crying by fathers. Second, the association between experienced childhood maltreatment and white matter integrity was not significant in whole‐brain analyses. Lastly, we found that the association between maltreatment exposure and excessive handgrip force during infant crying was absent in fathers with high tract integrity in the bilateral uncinate fasciculus. These findings possibly point to insufficient behavioral inhibition or emotional dysregulation in fathers who experienced childhood maltreatment, but buffering for this effect in those with larger integrity in brain fibers connecting the amygdala and prefrontal cortex.

## 1 INTRODUCTION

Paternal care greatly impacts the development of children; higher quality of care has been found to positively affect children's social, cognitive, and linguistic development (see Lamb, 2010, for reviews). Several neural factors such as brain structure and function have been shown to play an important role for the quality of paternal care (see Feldman et al., 2019; Rogers & Bales, 2019 for reviews). In females, experiences of childhood maltreatment have been found to affect both later parenting (see Norman et al., 2012; Van IJzendoorn et al., 2019) and brain structure (e.g. see Teicher & Samson, 2016 for a review).

To date, studies investigating the influence of experiences of childhood maltreatment on *paternal* brain and behavior are rare, even though their participation in childcare has substantially grown in modern western societies (Bakermans‐Kranenburg et al., 2019). Moreover, parenting researchers have recently been calling for a shift in attention toward biobehavioral models of fatherhood as the underlying mechanisms of paternal care remain relatively unknown and likely developed along different evolutionary pathways than those of mothers (Bakermans‐Kranenburg et al., 2019; Saxbe, 2017). In particular, studies examining responses to infant crying by fathers with varying degrees of experienced childhood maltreatment are needed because responses to infant signals are a crucial component of the parent–child relationship (Leerkes et al., 2011). Also, child maltreatment is still prevalent worldwide (Stoltenborgh et al., 2015) with emotional maltreatment being particularly prevalent in many countries (Gilbert et al., 2009; Stoltenborgh et al., 2012).

In the current study, we therefore examined the relation between fathers’ childhood maltreatment experiences (i.e. emotional and physical abuse and neglect) and their current behavioral responses to infant cry sounds (i.e. handgrip force). Additionally, we explore if this association is mediated or moderated by fathers’ brain structure (i.e. white matter integrity). Our focus lies on a particularly important but understudied area of parenting; the perinatal period, a period in which the foundation for postnatal parenting is build (e.g. Cabrera et al., 2008; Hechler et al., 2019; Lucassen et al., 2015; Witte et al., 2019).

Infant crying is a highly salient stimulus aimed at motivating a caregiver to provide appropriate parental care and consequently stop the infant's distress (Soltis, 2004). However, infant crying also holds the potential for triggering child abuse and neglect because it can onset feelings of anxiety, aversion, or anger in parents (e.g. Out, Bakermans‐Kranenburg, Van Pelt, & Van IJzendoorn, ; Reijneveld et al., 2004, Soltis, 2004). Having endured childhood maltreatment in one's past has been documented to be associated with emotional dysregulation throughout later life (e.g. Dvir et al., 2014; Pears & Capaldi, 2001). In turn, emotional dysregulation is thought to be detrimental for effectively managing emotional and distressing situations (e.g. see Mikulincer & Shaver, 2008; Reijman et al., 2016 for reviews), such as soothing a crying infant. Adults who experienced more childhood maltreatment show more negative behavioral responses to child signals and respond with harsher reactions when exposed to infant crying (Buisman et al., 2018, 2019).

Commonly found deficits in inhibitory and affective control in maltreated individuals (e.g. see Cowell et al., 2015; Dvir et al., 2014 for reviews) might cause these individuals to interpret infant signals as relatively negative and call upon time and energy consuming cognitive strategies to provide appropriate responses. This cognitive control may break down in cases of unconscious decision making or when under time pressure, such as is the case in responding to infant crying. Although all adults experience some level of physiological arousal when exposed to infant crying (e.g. Groh & Roisman, 2009; Out, Pieper, Bakermans‐Kranenburg, & Van IJzendoorn, 2010), men tend to find infant crying particularly aversive (Zeifman, 2003). Fathers have however been included in very few studies examining possible biological mechanisms underlying the relation between childhood maltreatment and responses to infant signals (but see Buisman et al., 2018; for a meta‐analytic comparisons of male vs. female brain reactivity to infant crying, see Witteman et al., 2019). As men now participate more in childcare and an increasing number of studies confirm their influence on child development (e.g. see Lamb, 2010), a better understanding of (biological) factors contributing to their ability to provide appropriate care is needed.

The handgrip dynamometer paradigm is used in parenting research as a behavioral marker of the ability to modulate a behavioral response (i.e. handgrip force) while being exposed to infant crying. Typically, females with insecure attachment representations (often resulting from experiencing suboptimal parenting) as well as parents at risk for perpetrating child abuse experience more hostile feelings and irritation while listening to infant crying and use more excessive handgrip force than females with secure attachment representations and parents at low risk for perpetrating child abuse (Crouch et al., 2008; Riem et al., 2012). Similarly, maltreating mothers used more excessive handgrip force than non‐maltreating mothers while listening to both infant crying and laughter (Compier‐de Block et al., 2015). Notably, males and females with experiences of childhood maltreatment (i.e. parental neglect) were found to have more difficulty modulating handgrip force when exposed to infant crying even though they did not rate the sound more negatively (Buisman et al., 2018). Combined, these studies call for a closer look at how fathers’ experiences of childhood maltreatment relate to maladaptive responses to infant signals. In particular, fathers’ functioning in the peripartum is of interest as this is a formative time that poses high demands on parents’ mental and physical health but is also crucial for successful adjustment to parenthood (e.g. see Saxbe et al., 2018). Moreover, exposure to infant crying, and the incidence of shaken baby syndrome, is especially frequent in the early postpartum (e.g. see Barr et al., 2006).

Connective white matter fibers are sensitive to adverse experiences throughout childhood and have, therefore, been a focal point in investigating the biological effects of childhood maltreatment (e.g. see Teicher & Samson, 2016 for a review). Since Teicher and Samson concluded in 2016 that childhood maltreatment likely affects white matter tracts of the dorsolateral prefrontal and orbitofrontal cortex, anterior cingulate, hippocampus, and corpus callosum (CC), several studies have added findings on the relation between childhood maltreatment exposure and white matter structure in young healthy individuals in particular. These studies confirmed the existence of a relation between childhood maltreatment and fibers connecting prefrontal and occipital areas in general (Ohashi et al., 2017), in the fronto‐occipital fasciculi (FOF; Lim et al., 2019; McCarthy‐Jones et al., 2018; Meinert et al., 2019), and in the longitudinal fasciculi specifically (LF; Lim et al., 2019; Meinert et al., 2019; Tendolkar et al., 2018). Findings of atypical structure in fibers connecting the orbitofrontal cortex and temporal regions (e.g. the uncinate fasciculus [UF]) were also confirmed in several studies (McCarthy‐Jones et al., 2018; Meinert et al., 2019; Ohashi et al., 2017). Four recent studies confirmed structural changes in the cingulum or in fibers connecting the cingulum with other brain regions (Kim, et al., 2019; McCarthy‐Jones et al., 2018; Meinert et al., 2019; Tendolkar et al., 2018), and in the CC (Jensen et al., 2018; Lim et al., 2019; McCarthy‐Jones et al., 2018; Meinert et al., 2019). Only one study so far confirmed a relation between childhood maltreatment and hippocampal projections (McCarthy‐Jones et al., 2018). An overview of samples, maltreatment measures, imaging parameters, tract delineation, and performed analyses in these studies has been presented in Table 1. In summary, consensus seems to be emerging on which white matter tracts are affected by exposure to childhood maltreatment in healthy adults. However, no studies so far have looked at possible functional outcomes related to these changes in white matter integrity that might be relevant for parenting behaviors.

**TABLE 1:** Overview of recent studies on associations between experienced maltreatment and white matter integrity in healthy youth and adults

	Sample	DWI acquisition	Tract delineation	Maltreatment	Analysis
Ohashi et al. (2017)^a^	262 healthy young adults	3T; 72 directions; *b* = 1,000 s/mm^2^; TE = 81 ms; TR = 6,000 ms; voxel size = 1.8 mm × 1.8 mm × 3.5 mm	Tractography	Categorical analysis of Maltreatment and Abuse Chronology of Exposure scale^b^	Graph Theory Analysis (e.g. number of fiber streams between groups)
Jensen et al. (2018)^a^	393 healthy young men	3T; 30 directions; *b* = 1,200 s/mm^2^; TE = 87 ms; TR = cardiac gated; voxel size = 2.4 mm isotropic	Tractography	Continuous analysis of prenatal and early life stress via maternal report, and adolescent stressful life events via self‐report	Correlational analysis between maltreatment and FA, MD, MTR and MWF in the genu and splenium, and in global (lobar) WM
Tendolkar et al. (2018)	120 healthy young men	1.5T; 34 directions; *b* = 1,000 s/mm^2^; TE = 98 ms; TR = 8,000 ms; voxel size = 2.5 mm isotropic	TBSS	Continuous analysis of Childhood Trauma Questionnaire^c^	Whole‐brain correlational analysis between maltreatment and FA and MD
McCarthy‐Jones et al. (2018)^a^	147 healthy middle‐aged adults	1.5T; 64 directions, *b* = 1,000 s/mm^2^; TE = 88 ms; TR = 8,400 ms; voxel size = 2.4 mm isotropic	TBSS	Continuous and categorical analysis of Childhood Adversity Questionnaire^d^	Correlational analysis between maltreatment and FA, FAT, and FW in ROIs
Lim et al. (2019)	18 maltreated youth, 18 psychiatric control youth, and 25 healthy control youth	3T; 32 directions; *b* = 1,300 s/mm^2^; TE = 104.5 ms; TR = cardic gated; voxel size = 2.4 mm isotropic	Tractography and TBSS	Continuous and categorical analysis of Childhood Trauma Questionnaire^c^	ANCOVA including streamline count, tract volume, FA, MD and RD; and whole‐brain correlational analysis
Meinert et al. (2019)	MNC cohort: 186 depressed adults and 210 healthy control adults MACS cohort: 397 (previously) depressed adults and 462 healthy control adults	MNC cohort: 3T; 20 directions; *b* = 1,000 s/mm^2^; TE = 95 ms; TR = 9,473 ms; voxel size = 1.8 mm × 1.8 mm × 3.6 mm MACS cohort: 3T; 2 × 0 directions; *b* = 1,000 s/mm^2^; TE = 90 ms; TR = 7,300 ms; voxel size = 2.5 mm isotropic	TBSS	Continuous analysis of Childhood Trauma Questionnaire^c^	Whole‐brain correlational analysis between maltreatment and FA, MD, RD, and AD
Kim, et al. (2019)	46 healthy young adults	3T; 30 directions for each *b*‐value; *b* = 1,000, 1,500, 2,000; TE = 94.2 ms; TR = 5,520 ms; voxel size = 2 mm isotropic	Tractography	Continuous analysis of Verbal abuse questionnaire^e^	Partial least square regression including maltreatment and connectivity likelihood maps based on ROIs

Note

Studies listed here appeared in a systematic literature search performed on Web of Science on January 17, 2020 using the search terms (TS = [white matter* OR DTI OR DWI OR fractional anisotropy OR diffusion tensor* OR structural integrity] AND TS = [maltreat* OR abus* OR neglect* OR early life stress] NOT TS = [visual neglect OR substance abuse]) AND LANGUAGE: (English) Indexes = SCI‐EXPANDED, SSCI, A&HCI, ESCI Timespan = from 2012.

Abbreviations: AD, axial diffusivity; FA, fractional anisotropy; FAT, free‐water corrected fractional anisotropy; FW, free‐water; MD, mean diffusivity; MTR, magnetization transfer ratio; MWF, myelin water fraction; RD, radial diffusivity; ROI, region of interest; T, tesla; TBSS, tract‐based spatial statistics; TE, echo time; TR, repetition time; WM, white matter.

^a^ These studies did not appear in the literature search but were added after a manual search of the literature.

^b^ Teicher, M. H., & Parigger, A. (2015). The “Maltreatment and Abuse Chronology of Exposure” (MACE) scale for the retrospective assessment of abuse and neglect during development. *PLoS ONE*, *10*, e0117423. http://dx.doi.org/10.1371/journal.pone.0117423.

^c^ Teicher, M. H., Samson, J. A., Rosenman, S., & Rodgers, B. (2004). Childhood adversity in an Australian population. *Social Psychiatry and Psychiatric Epidemiology*, *39*, 695–702.https://doi.org/10.1007/s00127‐004‐0802‐0

^d^ Bernstein, D. P., & Fink, L. (1998). *Childhood Trauma Questionnaire: a retrospective selfreport manual*. San Antonio (TX): The Psychological Corporation.

^e^ Teicher, M. H., Samson, J. A., Polcari, A., & McGreenery, C. E. (2006). Sticks, stones, and hurtful words: relative effects of various forms of childhood maltreatment. *American Journal of Orthopsychiatry*, *163*, 993–1000.https://doi.org/10.1176/appi.ajp.163.6.993

A growing body of literature suggests that atypical neural structure and function resulting from received parental care underlie parenting‐related responses in mothers (e.g. Kim et al., 2010; Mielke et al., 2016). This is not surprising since atypical white matter structure has been found to play an important role in cognitive and emotional difficulties as well as psychopathology among maltreatment survivors (e.g. Riem et al., 2019). Although fathers have not yet been investigated directly, atypical brain structure associated with childhood maltreatment has been found to mediate detrimental functional outcomes such as psychopathology and vulnerability to later life stress in both young males and females (Gorka et al., 2014; Hanson et al., 2015). Speculatively, the documented risk of intergenerational transmission of child maltreatment (e.g. see Buisman et al., 2020; Van IJzendoorn et al., 2019) may therefore rest in part on the neurobiological consequences of child maltreatment and the cognitive and emotional dysfunction resulting from these changes in brain structure and function, potentially interfering with nurturing, timely and appropriate parental responses.

Based on the available literature, we suggest that the association between experienced maltreatment and fathers’ ability to modulate handgrip force in reaction to infant crying might be mediated by the brain's white matter structure in regions commonly associated with childhood maltreatment. Alternatively, brain structure might play a moderating role leaving individuals more or less susceptible to cognitive and emotional dysfunction after experiencing maltreatment depending on their biological make‐up (e.g. see McCrory et al., 2010 for a review on the moderating role of genetics on the effects of maltreatment). Moreover, in infants, white matter structure has been found to moderate environmental influences on their negative emotional reactivity (Nolvi et al., 2020). Since few studies are available on a possible moderating role of white matter on behavioral outcomes relevant for parenting, we will merely explore (i.e. test without specific hypotheses) this moderation model.

Primarily, we hypothesize that higher levels of exposure to childhood maltreatment (i.e. emotional and physical abuse and neglect) are associated with more use of excessive handgrip force in response to infant crying in new and expectant fathers. Second, we hypothesize that white matter integrity in structures potentially affected by childhood maltreatment (the FOF, LF, UF, cingulum projections, and CC) mediates this relation. Lastly, we will explore the alternative model that white matter integrity in these structures moderates the relation between experienced childhood maltreatment and handgrip force in response to infant crying.

## 2 METHODS

### 2.1 Participants

First‐time expectant (*N* = 117) and new fathers (*N* = 136) were assessed for eligibility after reacting positively to recruitment invitations distributed via midwives, municipal records, infant welfare centers, and (online) advertisements. Fathers had to cohabitate with their partners who also did not have any previous children, speak Dutch, and were screened and excluded for MRI contraindications (e.g. metallic foreign objects, claustrophobia). Also, fathers were screened and excluded for current endocrine, psychiatric, or neurological disease, current or past serious head injury, use of soft drugs (e.g. cannabis) on a regular basis or hard drugs (e.g. cocaine, heroin) more than once within 3 months prior to participation, current heavy smoking or drinking, and use of (psychotropic) medication potentially interfering with neural measurements. Additionally, expectant fathers had to meet several other requirements due to an intervention taking place after the measurements reported here. Partners had to have an uncomplicated pregnancy of a singleton with a pregnancy duration of 18–31 weeks at the time of inclusion. Expectant fathers were excluded when their partners used alcohol, tobacco, or illicit drugs during the pregnancy or had a BMI over 30 before pregnancy. Additionally, participants were excluded when abnormalities were found during the medical 20‐week ultrasound examination, or in case of known birth defects in the families of either parent that caused excessive worry for the current pregnancy. For new fathers, the infants had to be full‐term (i.e. born after 37 week gestation), healthy, and around 2–4 months of age at time of inclusion. Additionally, new fathers were excluded if they used a baby carrier more than 5 hr a week due to an intervention taking place after the measurements reported here. One infant was born at 36 weeks and 6 days, but was considered healthy and therefore included. One father was not the biological father of the infant, but he had been cohabitating with the mother since mid‐pregnancy. Three participants reported a past diagnosis of depression, and one participant reported a diagnosis of a past anxiety disorder. All diagnoses occurred between 2 and 10 years before inclusion into the study. They did not have a current psychiatric diagnosis and did not currently use psychotropic medications and were therefore included in the analyses here.

As a result, 121 first‐time fathers with diffusion tensor imaging (DTI) data available were included into the analyses reported here, see Figure S1 for a flow chart. Of these 121 participants, handgrip data were missing for three participants due to time constraints during the lab session or technical difficulties, and information based on questionnaires was missing for five participants. Independent *t*‐tests indicated that new and expectant fathers did not differ in age, but there was a difference in the years of education following primary education; new fathers studied fewer years (*M* = 8.29, *SD* = 1.90) than expectant fathers (*M* = 8.98, *SD* = 1.30; *t*(108.27) = 2.37, *p* = .02). Kolmogorov–Smirnov test indicated that the education scores were not normally distributed (*p* = .00) and the distribution was skewed (i.e. skewness = 5.81). A chi‐square test indicated that country of birth (i.e. the Netherlands or other) did not differ between new and expectant fathers. See Table 2 for more sample characteristics.

**TABLE 2:** Demographic information of the sample

	*M* (*SD*)/*N* (%)	Range
Participant age (years, *N* = 121)	33.03 (4.09)	25–50
Education (years past primary education, *n* = 120)	8.62 (1.67)	3–10
Country of birth (*n* = 120)		
The Netherlands	114 (95%)	
Other	6 (5%)	
Handedness (*N* = 121)		
Right	107 (88%)	
Left	12 (10%)	
Ambidexter	2 (2%)	
Infant or fetal age (weeks)		
Gestational age (*n* = 57)	24.99 (2.77)	20–31
Infant age (*n* = 64)	11.24 (3.03)	7–21
Fetal sex (*n* = 57)		
Male	18 (32%)	
Female	28 (49%)	
Unknown	11 (19%)	
Infant sex (*n* = 64)		
Male	35 (55%)	
Female	29 (45%)	

Note

Information on education and country of birth is missing for one participant as he dropped out of study between the laboratory session and filling out online questionnaires.

Participants received financial compensation for each laboratory visit; €25 (plus travel allowance) for the visit described here. Participants received an extra €10 after the completion of the study if they completed at least 80% of the questionnaires. The study was approved by the Ethics Committees of the Leiden University Medical Centre and of the Department of Education and Child Studies at Leiden University. The study was carried out in accordance with the declaration of Helsinki and all participants gave informed consent.

### 2.2 Procedure

Participation in the study started with the visit to the laboratory described here. Participants were instructed to abstain from alcohol and excessive physical activity during the 24 hr before the visit, and from caffeine on the day of the visit. The visit started with instructions about the visit, after which saliva collection (for hormone measurements) took place. Next, participants took care of an infant simulator or their own infant for 10 min for the observation of their parenting behavior, during which the doll was programmed to cry uncontrollably for 5 min. This was followed by another saliva sample collection, after which participants underwent a brief training of the upcoming functional magnetic resonance imaging (fMRI) tasks on a laptop, which familiarized them with the tasks. A resting‐state scan, and a fMRI paradigm (using cry stimuli), as well as a fMRI task using video vignettes preceded the DTI scan described here. The neural measurements were followed by the handgrip cry paradigm. In the week following the visits, participants completed an online questionnaire. This questionnaire included basic demographics, the Love Withdrawal subscale of the Children's Report of Parental Behavior Inventory (CRPBI, Beyers & Goossens, 2003; Schludermann & Schludermann, 1983), the Conflict Tactics Scale—Parent Child (CTS; Straus et al., 1998), and the Edinburgh Postnatal Depression Scale (EPDS; Cox et al., 1987).

### 2.3 Measures

#### 2.3.1 Neural assessment—DTI

##### Data acquisition

Diffusion tensor imaging data were collected on a Philips 3.0 T Achieva MRI scanner (Philips Medical Systems, Best, The Netherlands) using a multi‐band single‐shot echo‐planar imaging sequence and an eight‐channel SENSE (Sensitivity Encoding) head coil. The following scan parameters were used: repetition time = 2,777 ms, echo time = 98 ms, flip angle = 90°, *b*‐value = 2000 s/mm^2^, voxel dimensions = 2 mm isotropic, number of slices = 57, and no slice gap. DTI data were acquired along 80 directions, together with nine images having no diffusion weighting (*b* = 0), scanned in posterior–anterior direction. One additional reversed *b* = 0 scan was made in anterior–posterior direction. The total scanning time was 4.47 min.

##### Data preprocessing

The Oxford Centre for Functional MRI of the Brain software library (FMRIB's Software Library version 6.0; Smith et al., 2004) was used to preprocess and analyze DTI data. First, *topup* was run because data were collected with reversed phase‐encode blips, resulting in pairs of images with distortions going in opposite directions. From these pairs, the susceptibility‐induced off‐resonance field was estimated using a method similar to that described in Andersson et al. (2003). To run *topup*, the number of slices was reduced to 56 by removing the most inferior slice. Next, *topup* combined two images (i.e. the first *b* = 0 volume from the DWI dataset and the reversed *b* = 0 scan) into a single file which was used to estimate the susceptibility field. Then, all non‐brain material was extracted using *BET* (Brain Extraction Tool, Smith, 2002). Second, diffusion data were corrected for eddy current‐induced distortions and subject movements using *eddy* (Andersson & Sotiropoulos, 2016). Outliers were replaced within eddy using the *repol* option (Andersson et al., 2016). We visually examined corrected diffusion images, and also obtained quantitative metrics relating to image quality using FSL's *QUAD* (QUality Assessment for DMRI) and *SQUAD* (Study‐wise QUality Assessment for DMRI) tools (Bastiani et al., 2019). Third, fractional anisotropy (FA) images were created by fitting a tensor model to the raw diffusion data using *dtifit* from the FMRIB's Diffusion Toolbox. From these maps, FA was calculated. Fourth, to reduce partial volume effects and registration misalignments, *TBSS* (Tract‐Based Spatial Statistics; Smith et al., 2006) was used. All subjects' FA data were aligned into a 1 × 1 × 1 mm standard space (i.e. FMRIB58_FA template) using the nonlinear registration tool *FNIRT* (Andersson et al., 2007; Smith et al., 2004), which uses a b‐spline representation of the registration warp field (Rueckert et al., 1999). Next, the mean FA images were created and thinned to create a mean FA skeleton which represents the centers of all tracts common to the group. After visual inspection of our data, we set a threshold of 0.3 for the average of all aligned FA images to reduce inter‐subject variability when creating a white matter skeleton. Each subject's aligned FA data were then projected onto this skeleton and the resulting data fed into voxel‐wise cross‐subject statistics. Additionally, mean diffusivity (MD) images were also created and fed into TBSS.

##### DTI data analysis

To test for statistically significant associations between maltreatment scores and FA or MD values, we employed nonparametric permutation testing using maltreatment scores as a covariate. Voxel‐wise permutation‐based analyses (Winkler et al., 2014) were performed using *randomise* with 5,000 permutations. Images were corrected for multiple comparisons using *TFCE* (Threshold‐Free Cluster Enhancement; Smith & Nichols, 2009), and significance was determined using the 95th percentile of the null distribution of permutated input data of the maximum *TFCE* scores, allowing to correct estimated cluster sizes for family‐wise error. For the mediation analysis, individual FA and MD values for any significant clusters were extracted using *cluster* and *fslmaths* functions. For the moderation analysis, anatomical skeletonized masked were created using *fslmaths* and the JHU White‐Matter Tractography Atlas (Hua et al., 2008; Mori et al., 2005; Wakana et al., 2007) for the CC (including the genu, body, and splenium; label numbers 3–5), bilateral cingulum (including both the gyrus and hippocampal projections, label numbers 35–38), bilateral sagittal stratum (including the inferior LF and inferior FOF, label numbers 31–32), bilateral superior FOF (label numbers 43–44), bilateral superior LF (label numbers 41–42), and bilateral UF (label numbers 45–46). Individual FA values were then extracted using *fslmeants* and used in further analyses. Visual inspection indicated that all ROIs contained voxels that survived the FA threshold. All extracted variables were normally distributed.

#### 2.3.2 Behavioral assessment—handgrip paradigm

##### Procedure

Participants were exposed to infant crying and images representing either their own or an unknown infant while they were asked to squeeze a handgrip dynamometer. During the task, participants were seated in front of a computer screen wearing headphones while holding a dynamometer in their dominant hand. During an initial training period without cry sounds or images, participants were asked to alternate between squeezing the handgrip dynamometer at full and half strength while they received visual feedback from a monitor indicating the strength they used. Once participants could accurately alternate between full and half strength (half strength being 50% of the strength used at full strength), the monitor was turned away and the actual task began without feedback on performance.

##### Task images

To create suitable own infant images for this task, new fathers either provided a full‐color digital photo of their infant's face with a neutral expression prior to the visit, or a picture was taken at the beginning of the visit. New fathers’ own infant images were overlain with a black face contour mask using Adobe Photoshop CS removing all background features and resized to 640 × 480 pixels. Expectant fathers either provided a full‐color digital photograph of themselves prior to the first visit, or a picture was taken at the beginning of the visit. The expectant participant's picture met the following criteria: it showed their face, en face, with a neutral expression, a light and neutral background, without piercings, make‐up, or glasses. Expectant fathers’ photographs were edited using Adobe Photoshop CS to remove unwanted facial features (e.g. facial hair). Subsequently, morphed images representing participant's own infant were created by combining 75% of an average infant image (created by the authors of Hahn et al., 2015, from 10 female and 10 male infant faces) and 25% of participant's own picture, using Fantamorph 5 Deluxe (www.fantamorph.com). These morphed images based on their own picture were overlain with a black face contour mask removing all background features and resized to 640 × 480 pixels. Participants were familiarized with their edited own infant image (i.e. picture of their own infant for new fathers and morphed images based on their own picture for expectant fathers) before onset of the task. Additionally, expectant fathers were told that a future infant of theirs might look similar to this image. To create suitable “unknown infant” images, the same protocol was used. This resulted in a masked and resized picture of a real but unknown infant used for new fathers, and a masked and resized morphed image of an infant for expectant fathers (i.e. 75% of the average infant image morphed with 25% of a male unknown to the participants). The decision to include images of “own” and “unknown” infants was based on the idea in evolutionary psychology that perceived genetic relationships influence paternal behavior, with closer genetic relationships supposedly enhancing paternal investment. This is considered particularly relevant in assessing the effects of threat to infant (see Van't Veer et al., 2019) and the effects of vasopressin on handgrip force (see Alyousefi‐van Dijk et al., 2019). The distinction between own and unknown infants was expected to be nonrelevant for the analyses reported here.

##### Task sounds

To create suitable sounds for this task, a total of six cry sounds were recorded from six infants (three males, three females), using a TasCam DR‐05 solid state recorder with at a 44.1 kHz sampling rate and 16 bit. All sounds were recorded between 2 days and 5.5 months postnatally. All cry sounds were scaled, the intensity is normalized to the same mean intensity (74 Db) and sounds are edited to last for 10 s using PRAAT software (version 6.0.37; Boersma & Weenink, 2018). For each cry sound, a neutral auditory control stimulus was created by calculating the average spectral density over the entire duration of the original sound. A continuous sound of equal duration was re‐synthesized from the average spectral density and amplitude modulated by the amplitude envelope, extracted from the original sound. After this procedure, all auditory stimuli and control stimuli were intensity matched. The neutral auditory control stimuli were identical to the original auditory stimuli in terms of duration, intensity, spectral content, and amplitude envelope.

##### Task design

The procedure used here was identical to that used in a similar (but not the same) sample (Alyousefi‐van Dijk et al., 2019; see also Alyousefi‐van Dijk et al., 2020). The task was administered using E‐Prime software (version 2.0; Psychology Software Tools, Inc.). Squeeze intensities (in kg) were transferred directly from the dynamometer to AcqKnowledge software (version 4.3.1; Almond, 2017). First, a baseline measure of three maximum strength trials, each followed by half strength trials, was administered. Then, four conditions of three max‐half trials were presented in semi‐random order; (a) viewing an image of own infant while hearing control (scrambled) sounds (Own Neutral); (b) viewing an image of own infant while hearing cry sounds (Own Cry); (c) viewing an image of an unknown infant while hearing control (scrambled) sounds (Other Neutral); and (d) viewing an image of an unknown infant while hearing cry sounds (Other Cry). Sounds and images were presented throughout each trial lasting 12 s. Eight seconds after the beginning of each trial, participants were prompted to squeeze maximally (instructions displayed for 1s). After an interval of 2s, participants were prompted to squeeze at half strength (instructions were displayed for 1s). A fixation cross was shown for 3s between each trial.

##### Scoring

Similar to previous studies (e.g. Alyousefi‐van Dijk et al., 2019; Bakermans‐Kranenburg et al., 2012; Compier‐de Block et al., 2015; Riem et al., 2012), grip strength modulation was calculated by dividing half‐strength squeeze intensity by the preceding full‐strength squeeze intensity, meaning that scores of over 0.50 indicated excessive force on the half‐strength squeeze attempt. MATLAB (version 8.0.0.783; Mathworks) was used to identify peak intensities for each squeeze. Trials believed to represent measurement errors (i.e. ratios <0 or >2) were disregarded. Handgrip force measures were found to be reliable for the four conditions (*α*_range_ = 0.75–0.83). Therefore, the three trials per condition were averaged as indicators of handgrip force in each condition. Since the distinction between own and unknown infants was not relevant for the analyses described here, and the conditions with own or unknown images showed high correlations (*r* = 0.72 for neutral sounds and *r* = 0.79 for cry sounds), the own and unknown infant trials were taken together. Mean handgrip force ratios (*n* = 118) were roughly around the intended 0.5 for both the control (*M* = 0.59, *SD* = 0.14, range 0.22–0.95) and the infant cry sounds (*M* = 0.58, *SD* = 0.14, range 0.23–0.96). To create one value representing the cry‐control sound contrast, a residualized score was calculated by residualizing the squeeze during cry trials for the squeeze during control trials (identical to Alyousefi‐van Dijk et al., 2020). Residualized scores were created to avoid issues associated with difference scores (MacKinnon, 2012).

One outlier (i.e. *Z* score > 3.29) was detected in the residualized scores, and this value was winsorized by adding the difference between the second and third largest values to the second largest value, and replacing the outlier with this new value. Residualized scores were found to be distributed normally, and did not differ between new and expectant fathers (*t*(116) = 1.31, *p* = .19).

#### 2.3.3 Self‐reported child maltreatment

Identical to the procedure in a similar (but not the same) sample (Thijssen et al., 2018), participants completed seven items from the Withdrawal of Relations subscale of the Children's Report of Parental Behavior Inventory (CRPBI, Beyers & Goossens, 2003; Schludermann & Schludermann, 1983) of which two items were slightly adapted for a smoother translation. To obtain a more comprehensive measurement of parental love‐withdrawal and emotional maltreatment, the questionnaire was complemented with four items from the Parental Discipline Questionnaire (Patrick & Gibbs, 2007, see Huffmeijer et al., 2011 for the resulting scale). The resulting scale contained 11 items such as “My mother/father is a person who, when I disappoint her/him, tells me how sad I make her/him.” Participants rated how well each of the statements described their mother's and father's behavior separately on a 5‐point scale ranging from 1 = “not at all” to 5 = “very well.” Two participants reported that one parent had not been present throughout their childhood and scores for this particular parent where therefore not taken into account. A mean parental love‐withdrawal score was computed by averaging the highest scores per item, being either that reported about the participant's father or mother. Scores per item were roughly 50% of the times highest for father and for mother, except for item 7 (i.e. “When I disappointed my parent he/she told me how sad I made him/her”), where the score for mother was more often the highest. Additionally, participants completed the Conflict Tactics Scale—Parent Child (CTS, Straus et al., 1998). We used items from the subscales Psychological aggression, Minor physical assault, Severe physical assault, and Neglect, resulting in a total of 18 items. Items were answered on a 7‐point scale (0 =”never,” 1 = “once,” 2 = “twice,” 3 = “3–5 times,” 4 = “6–10 times,” 5 = “11–20 times,” 6 = “more than 20 times”). Averaging scores on the minor and severe physical assault scales resulted in a Physical assault score, which combined with the scores on Psychological aggression formed an Abuse score. An overall CTS score was computed by averaging the Abuse and Neglect scales. Both maltreatment questionnaires were found to be reliable (*α* = 0.85 for CTS and *α* = 0.90 for CRPBI). Average maltreatment scores (*n* = 116) were 0.75 (*SD* = 0.75) for the CTS and 2.02 (*SD* = 0.78) for the CRPBI, range_CTS_ = 0–3.48 and range_CRPBI_ = 1–4.18. Additionally, we found good reliability for the combined CTS and CRPBI item scores (*α* = 0.89), and therefore combined the standardized total scores of both questionnaires into one average maltreatment score, which was used in all further analyses.

A Kolmogorov–Smirnov test indicated that the combined standardized maltreatment average was somewhat skewed (i.e. skewness = 5.47). Data were not transformed as maltreatment scores were the predictor in our model. Average maltreatment scores did not differ between new and expectant fathers (*t*(114) = 0.51, *p* = .61).

#### 2.3.4 Self‐reported perinatal depression

Participants filled out all 10 items of the Postnatal Depression Scale (EPDS; Cox et al., 1987), where a total score represents the sum of all symptoms present. The EPDS is a self‐report screening tool, devised to detect mild perinatal depression in the community. Previously, the EPDS has been shown to be suitable for the assessment of depression in the prenatal period (e.g. see Cox et al., 2014) and in fathers (e.g. Edmondson et al., 2010). The clinically relevant cutoff for EPDS scores in fathers is currently under debate, but it is believed to be lower than that for mothers, typically between 7 and 10 (e.g. Edmondson et al., 2010) but possibly even lower (Matthey et al., 2001). The EPDS questionnaire was found to be reliable in the sample described here (*α* = 0.74).

A Kolmogorov–Smirnov test indicated that the total EPDS score was somewhat skewed (skewness = 4.68). Data were not transformed as perinatal depression scores were a covariate in our model. Average EPDS scores (*n* = 116) were low (*M* = 4.55, *SD* = 0.28, range 1–14). Perinatal depression scores did not differ between new and expectant fathers (*t*(114) = 1.32, *p* = .19).

### 2.4 Imputation

After obtaining descriptive results for all variables, multiple imputation was applied. The majority of missing values were the results of participants not filling out the questionnaires, resulting in 4% of cases missing for experienced maltreatment and perinatal depression scores. Additionally, handgrip data were missing for three participants (3%). Educational score was missing for one participant (1%). Age, and FA and MD values in all ROIs were available for all participants. Missing values were imputed using multiple imputation where these values were estimated several times, resulting in several complete datasets. Missing values were imputed using the package *mice* (Van Buuren & Groothuis‐Oudshoon, 2010) in R (R Development Core Team, 2008). Specifically, *mice* imputes multivariate data by means of chained equations. In all further statistical analyses, all imputed datasets (i.e. 50) are analyzed, and the results are then combined using specific combination procedures based upon variability in the standard errors and *p* values of the imputed datasets. Predictive mean matching (Little, 1988) was used to guarantee that imputed values do not fall outside the range of the variable or outside the observed values of the variable. In all, 100 iterations were run. Autocorrelation function plots (Azur et al., 2011) were visually inspected and indicated that all imputations converged. Little's MCAR test was not significant (*χ* ^2^(26) = 19.09, *p* = .83), indicating that data were missing completely at random. Importantly, bivariate correlations between all variables were approximately the same in the imputed dataset (see Table 3) and in the non‐imputed dataset (see Table S1). Further analyses were conducted in SPSS version 25.

**TABLE 3:** Correlations of the pooled observed variables in the imputed dataset (*N* = 121). Pearson's correlations (*r*) are reported

	Age	Edu	EPDS	Maltr	HG	CC	Bi cingulum	Bi inf FOF LF	Bi sup FOF	Bi sup LF	Bi UF
Age		−0.04	−0.07	0.08	−0.05	−0.08	0.02	−0.06	−0.14	−0.19*	0.01
Edu			0.02	−0.17	0.16	−0.04	0.01	−0.02	0.00	−0.09	−0.07
EPDS				0.15	0.00	0.09	0.08	0.05	0.02	0.07	−0.12
Maltr					0.22*	0.04	0.00	0.12	−0.04	−0.10	0.01
HG						−0.05	0.02	−0.01	−0.07	0.08	0.00
CC							0.64**	0.57**	0.48**	0.57**	0.43**
Bi cingulum								0.52**	0.38**	0.49**	0.40**
Bi inf FOF LF									0.41**	0.45**	0.36**
Bi sup FOF										0.43**	0.26**
Bi sup LF											0.38**
Bi UF											

Abbreviations: Bi, bilateral; CC, corpus callosum; Edu, educational level; EPDS, Edinburgh Postnatal Depression Scale; FOF, fronto‐occipital fasciculus; HG, residualized handgrip force ratio; inf, inferior; LF, longitudinal fasciculus; Maltr, experienced childhood maltreatment; sup, superior; UF, uncinate fasciculus.

* *p* < .05 (two‐tailed).

** *p* < .01 (two‐tailed).

### 2.5 Statistical analyses

A mediation analysis was run on the imputed dataset to test individual FA or MD values in any significant clusters (i.e. clusters significantly associated with experienced maltreatment scores in whole‐brain analyses) as mediators for an association between experienced maltreatment scores and handgrip force during infant crying. Next, an exploratory moderation analysis was run on the imputed dataset to test moderation by individual FA values for the anatomical structures mentioned in the hypotheses on an association between experienced maltreatment and handgrip force. Perinatal depression, age, and education levels were used as covariates.

## 3 RESULTS

### 3.1 Association between childhood maltreatment and handgrip force

Childhood maltreatment scores showed a significant positive correlation with handgrip force in response to infant crying (i.e. *r*(119) = 0.22, *p* = .02; see Table 3), indicating that higher levels of maltreatment were related to more use of excessive force during infant crying in new and expectant fathers.

### 3.2 Association between childhood maltreatment and white matter integrity

No white matter clusters were found to relate significantly to childhood maltreatment scores in whole‐brain analyses (i.e. *p* > .05 corrected) for either FA or MD; thus, no mediation analysis was run. Likewise, there were no significant correlations between extracted FA values in the regions of interest (ROI) and experienced maltreatment, see Table 3.

### 3.3 Moderation effects of white matter integrity

**FIGURE 1:**
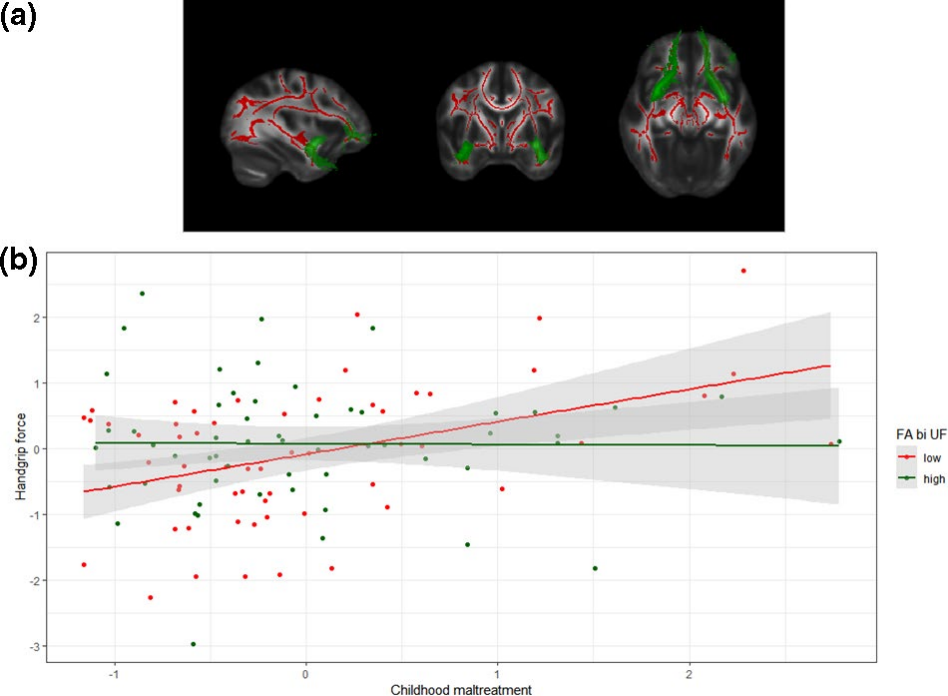
Mean skeletonized FA in the bilateral uncinate fasciculus (bi UF) moderates the association between experienced childhood maltreatment and handgrip force ratios in response to infant crying. (a) Skeletonized FA values are depicted in red, with an anatomical mask (JHU White‐Matter Tractography Atlas) for the bilateral UF depicted in green. (b) A significant two‐way interaction was observed such that the mean FA value in the bilateral UF moderated the association of experienced childhood maltreatment and handgrip force in response to infant crying. The significant predictive value of maltreatment on handgrip force was only present for those individuals with low (*N* = 58; depicted in red) rather than high (*N* = 56; depicted in green) FA levels in the UF. Visualization is based on the complete cases dataset

Individual mean skeletonized FA values structurally defined by the JHU White‐Matter Tractography Atlas were extracted (*N* = 121) for the CC (*M* = 0.65, *SD* = 0.00), bilateral cingulum (including both the gyrus and hippocampal projections; *M* = 0.50, *SD* = 0.00), bilateral sagittal stratum (including the inferior LF and inferior FOF; *M* = 0.51, *SD* = 0.00), bilateral superior FOF (*M* = 0.47, *SD* = 0.00), bilateral superior LF (*M* = 0.51, *SD* = 0.00), and bilateral UF (*M* = 0.46, *SD* = 0.00). The association between maltreatment and handgrip force was moderated by FA values in the bilateral UF, *B* = −8.11, *SE* = 3.02, 95% confidence interval = [−14.02, −2.19], *t*(114) = −2.69, *p* = .01. A closer look at this interaction effect revealed that participants with low FA values in the bilateral UF (based on a median split at 0.0009) showed a significant positive association between experienced child maltreatment and handgrip force (*B* = 0.52, *SE* = 0.15, 95% CI [.22, 0.82], *t*(56) = 3.41, *p* < .01), whereas no significant association was found between experienced child maltreatment and handgrip force for participants with high FA values in this structure (*B* = 0.09, *SE* = 0.16, 95% CI [−0.22, 0.40], *t*(55) = 0.57, *p* = .57), see Figure 1.

No significant moderation effects were found for the CC, bilateral cingulum, bilateral sagittal stratum, bilateral superior FOF, or bilateral superior LF. Findings of the moderation analyses were comparable with and without multiple imputation (see Table S2). Also, results were comparable in new and expectant fathers (i.e. confidence intervals in both groups overlapped with the overall results), see Tables S3 and S4.

## 4 DISCUSSION

In this study, we investigated the relation between childhood maltreatment experiences, handgrip force in reaction to infant crying, and white matter tract integrity in new and expectant fathers. As expected, we found that childhood maltreatment experiences were related to paternal behavioral responses; fathers with higher levels of experienced maltreatment used more excessive handgrip force while listening to infant crying. No significant relation was found between reported maltreatment and white matter integrity in the whole‐brain analysis. Therefore, white matter integrity did not mediate the relation between maltreatment and handgrip force. However, this relation was moderated by white matter integrity in the bilateral UF such that childhood maltreatment experiences were only predictive of handgrip force during infant cry exposure in fathers with low levels of white matter integrity in the bilateral UF.

In line with earlier findings in mothers and fathers (Buisman et al., 2018), we found that fathers’ experiences of childhood maltreatment were predictive of using more handgrip force during infant cry exposure. Speculatively, this finding might indicate that maltreated fathers experience more aversion and anxiety when presented with negative infant signals (e.g. Out, Pieper, Bakermans‐Kranenburg, Zeskind, et al., 2010), and are therefore more likely to respond harshly. Emotional dysregulation or deficient inhibitory control may underlie this phenomenon. Indeed, childhood maltreatment is known to be related to an attentional bias toward negative or threatening stimuli in both childhood and adulthood, possible serving as an adaptive mechanism preparing the individual for frequent exposure to threatening situations (e.g. Gibb et al., 2009). Also, neural hyperactivation in regions involved in threat‐detection in response to various emotional facial expressions (e.g., Van Harmelen et al., 2013) points to a mechanism of interpreting emotional expressions as highly salient and potentially dangerous in maltreated individuals (see also Oosterman et al., 2019; Pollak et al., 2000).

Parenting research supports this idea as mothers exposed to childhood sexual and/or physical abuse have been found to provide lower quality of care with their own child, and this parental quality has been found to relate to the anatomy of brain structures known to underlie cognitive (and not emotional) empathy (e.g. Mielke et al., 2016). Specifically, when given the instruction to inhibit prepotent motor responses, maltreated individuals show larger reaction times despite increased brain activation in areas responsible for inhibitory and response control, as compared to individuals who were not exposed to early life adversity (Mueller et al., 2010; Navalta et al., 2006). Childhood maltreatment has been found to impact on the effective connectivity of the neural inhibitory control network during a task in which prepotent motor responses had to be inhibited (Elton et al., 2014). Taken together, it is plausible that fathers with higher levels of experienced childhood maltreatment in our sample struggled to modulate handgrip force in response to infant crying because the stimulus was processed as particularly aversive and inhibitory control over their behavioral response was inadequate.

Contrary to our expectations, we did not find a direct relation between fathers’ childhood maltreatment experiences and white matter integrity in a whole‐brain approach. Previously, several studies examining continuous measures of maltreatment exposure (i.e. graduation of severity) in similar populations of healthy young adults have reported widespread associations between maltreatment and white matter integrity (e.g. Hanson et al., 2015; Jensen et al., 2018; Kim, et al., 2019; McCarthy‐Jones et al., 2018; Ohashi et al., 2017; Tendolkar et al., 2018) and several of these studies looked at similar types and levels of abuse and neglect as in the study reported here (e.g. Hanson et al., 2015; McCarthy‐Jones et al., 2018; Tendolkar et al., 2018). However, most of these studies only reported associations between maltreatment and specific ROIs. Notably, we also did not find correlations between self‐reported childhood maltreatment and commonly reported ROIs, indicating that the absence of findings in our whole‐brain analysis is probably not merely due to stringent corrections for multiple testing in this analysis. Only one other study reported whole‐brain white matter integrity in a similar sample size of healthy young men (*N* = 114), and no significant clusters were found when testing for an association with overall maltreatment severity (Tendolkar et al., 2018), although a significant reduction in white matter integrity for several areas (including the UF) was found when looking at physical neglect rather than a combined maltreatment score. Additionally, a recent large study examining whole‐brain white matter integrity in adult participants with or without major depressive disorder (*N* = 396) found several clusters to be significantly associated with maltreatment scores (i.e. emotional and physical neglect and abuse, as well as sexual abuse), irrespective of psychiatric diagnosis (Meinert et al., 2019). These authors concluded that the exact type of abuse is probably irrelevant and that an “overall contribution of early negative life events” seems to underlie these effects. Taken together, these findings raise an important question. Why do sufficiently powered studies such as Tendolkar et al. (2018) and the current study finds no significant association between DTI measures and overall maltreatment scores in whole‐brain analyses such as in Meinert et al. (2019)? Considering our sample size of *N* = 116, we were sufficiently powered (power 0.97) to detect the effect size reported by Meinert and colleagues (i.e. *β* = −0.343, CI −0.428, −0.252). More studies using unbiased whole‐brain approaches investigating an association between maltreatment severity and white matter integrity are warranted as the current consensus in literature seems to be based upon ROI studies while outcomes have not yet been reproduced within whole‐brain studies (see also Hart & Rubia, 2012 for a review). Possibly, a robust effect of aberrant white matter integrity associated with maltreatment exposure in whole‐brain analyses can only be found when comparing maltreated and non‐maltreated groups (see Lim et al., 2020 for a meta‐analysis).

Exploratively, we found that white matter integrity of the UF moderated the association between experienced maltreatment and fathers’ modulation of handgrip force during infant cry exposure, where the association between maltreatment and handgrip force was absent in fathers with higher tract integrity in the UF. Interestingly, the UF is known to support adequate inhibitory regulation of emotional reactivity or motor responses that depend on effective communication between the frontal and temporal structures (i.e. prefrontal cortex and amygdala; Depue et al., 2016; Versace et al., 2015). As such, the UF plays an important role in “valence‐based biasing of decisions” and in the incorporation of reward and punishment history into memory‐dependent processes (Von der Heide et al., 2013). Likewise, higher structural integrity of the UF has previously been shown to protect against the well‐documented relation between childhood maltreatment and later anxiety and stress resilience (Kim et al., 2019). Additionally, activity and connectivity of the prefrontal cortex has recently been found to be an important differential susceptibility marker for the effects of environmental influences on child development (Crone et al., 2020). Although further studies are needed to replicate and elaborate on this finding, we speculate that part of the dysfunctional effect of maltreatment on later parenting is dependent on structural integrity of the UF which is needed to effectively downregulate emotional hyperreactivity as well as support adequate inhibitory control over behavioral responses, particularly in reaction to negative stimuli. Hypothetically, maltreated fathers with high tract integrity in the UF might be protected against effects of childhood maltreatment on emotional hyperreactivity and impaired behavioral inhibition. If so, tract integrity in the uncinate fasciculus might be one of the underlying factors contributing to resilience in the intergenerational transmission of maltreatment.

Several limitations of the current study should be noted. First, the field of neurobiological aspects of paternal care is relatively unexplored territory and replication studies and meta‐analyses will be highly valuable in approximating valid conclusions. Although the current study was relatively well‐powered, more studies are needed to uncover the mechanisms and contributing elements of paternal care. Specifically, the moderation effect reported here was part of an exploratory analysis and therefore not corrected for multiple testing. Future studies could use these findings as hypotheses. Also, as our design was cross‐sectional, any conclusion about the origins of the relation between maltreatment history and later parenting remain speculative. Although there is a large body of literature supporting the mechanism proposed here, it should be noted that other explanations for our findings (e.g. genetic factors contributing to both maltreatment and excessive force in the handgrip task) cannot be not ruled out. Second, there is little agreement between prospective and retrospective reports of maltreatment (Baldwin et al., 2019). However, prospective measures (e.g. official CPS reports) are assumed to capture only the top of the proverbial iceberg, whereas retrospective measures (i.e. self‐reported child maltreatment) have been suggested to capture a broader range of maltreatment experiences (e.g. Baldwin et al., 2019; Smith et al., 2008). Additionally, some (e.g. Buisman et al., 2019), but not others (e.g. Meinert et al., 2019), have found that a distinction between maltreatment subtypes is important for the neurobehavioral sequelae. Shedding more light on this issue was outside the scope of this study, and the measures used were not equipped to provide reliable subscales of common maltreatment types. However, the potentially differential effects of various types of maltreatment in fathers should be explored in future studies. Third, in our moderation analyses, we used averaged values of the most commonly used indicator of white matter integrity (i.e. FA). Previous studies have provided indications that various white matter indicators relate differently to maltreatment history (e.g. Jensen et al., 2018; McCarthy‐Jones et al., 2018) and this should be studied further. Also, averaging skeletonized FA values within a (relatively) large ROI may have increased the chance of missing associations, leading to false negatives. Future studies looking into these associations could explore different methods, for example using small volume correction. Forth, our sample of relatively highly educated and low‐risk fathers is not representative of the general population. Socioeconomic status may be related to maltreatment history and parenting, or may moderate associations between maltreatment history and parenting. Studies including more diverse samples could investigate this. Fifth, we did not include real‐life parenting measures such as parenting sensitivity or involvement. Therefore, our speculations about the impact of the maltreatment‐related effects on handgrip force on paternal care are not tested directly in the same sample, even though the handgrip paradigm is a highly standardized and well‐controlled measure of reactions to infant signals. Ideally, future studies combine lower level mechanistic measures (e.g. brain structure and function, hormonal levels and reactivity, physiological responses) with parent–child observations, or include other measures more closely related to parenting quality and the effects thereof on children.

In conclusion, our results indicate that negative childhood caregiving experiences are associated with new and expectant fathers’ difficulties to modulate behavioral response to infant crying. Speculatively, infant crying was processed as particularly aversive by maltreated fathers and inhibitory control over their behavioral response was inadequate. Moreover, this effect was only present in fathers with low structural connectivity between the prefrontal cortex and the amygdala, that is, bilateral UF, a brain structure that supports effective downregulation of emotional hyperreactivity as well as adequate inhibitory control over behavioral responses to negative stimuli. Importantly, our findings indicate that the detrimental effects of child maltreatment history on parenting during exposure to infant distress are measurable in men shortly before and after the birth of their first child, a period in which exposure to infant crying is (about to be) common. As the perinatal period is particularly formative for new parents, our findings provide more insight into which fathers might be at risk of developing suboptimal behavioral reactions to infant distress signals. Future research could indicate how these findings relate to more direct measures of parenting (e.g. parenting sensitivity). In hopes of finding effective early intervening where needed, a more thorough understanding of fathers’ abilities to provide adequate care for their children, and all contributing factors, is needed.

## CREediT author statement

KA‐vD: Methodology, Formal analysis, Investigation, Data Curation, Writing—Original Draft, Visualization, and Project administration; NvdK: Software, Formal analysis, Investigation, Data Curation, and Writing—Review and Editing; RSMB: Methodology, Software, Formal analysis, Data Curation, Writing—Review & Editing, and Visualization; LIH: Software, Formal analysis, Investigation, Data Curation, and Writing—Review and Editing; AML: Investigation, Data Curation, Writing—Review and Editing, and Project administration; MMER: Methodology, Formal analysis, Writing—Review and Editing; CS: Methodology, Writing—Review and Editing, and Supervision; MHvIJ; Conceptualization, Methodology, Writing—Review and Editing, Supervision, and Funding acquisition; MJB‐K: Conceptualization, Methodology, Writing—Review and Editing, Supervision, and Funding acquisition.

## CONFLICT OF INTEREST

None.

## Supporting information

Figure S1

Table S1

Table S2

Table S3

Table S4

## Data Availability

The data that support the findings of this study are available from the corresponding author upon reasonable request.
