# Supplementary material for: White matter integrity moderates the relation between experienced childhood maltreatment and fathers’ behavioral response to infant crying
Source: Dev Psychobiol. 2020 Nov 17;63(5):1399–414. doi: 10.1002/dev.22058 (PMC8451806; doi:10.1002/dev.22058)
Supplement: Supplementary file 1 — Figure S1 [file DEV-63-1399-s004.docx]

**
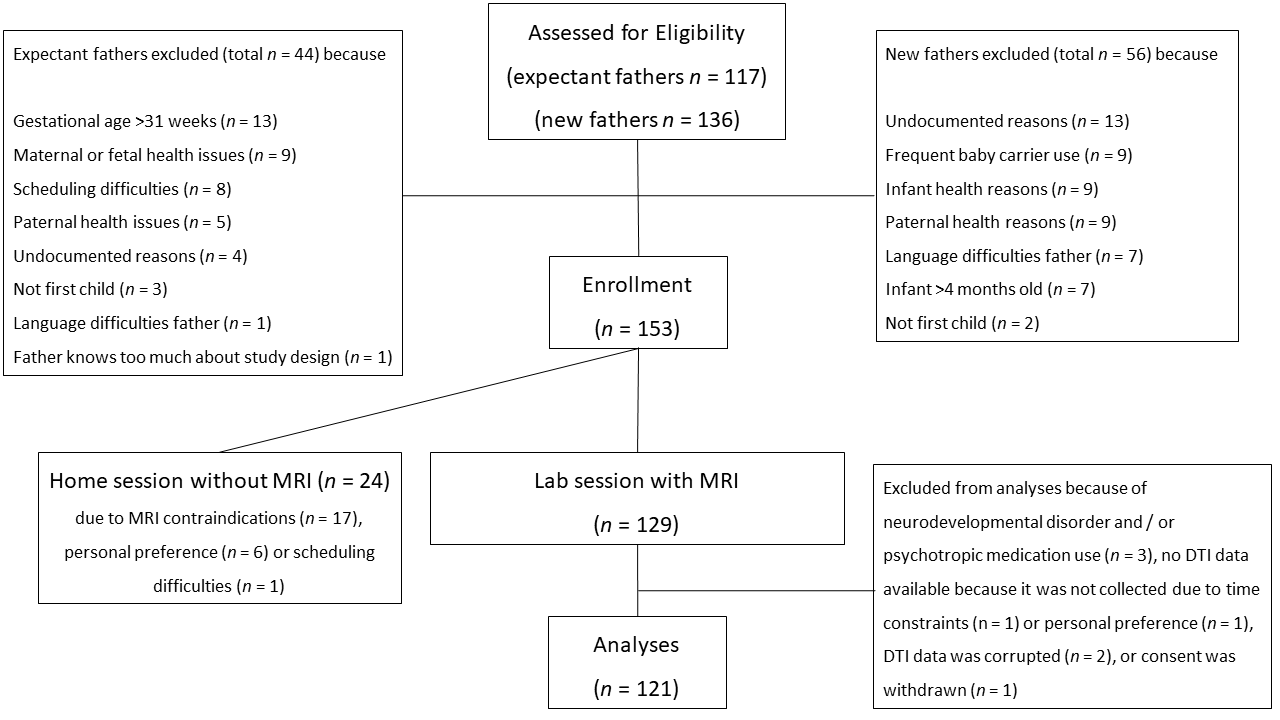
Supplementary Materials Figure 1.** Flow chart depicting numbers for participant enrolment, allocation, and data analysis.
