# Supplementary material for: White matter integrity moderates the relation between experienced childhood maltreatment and fathers’ behavioral response to infant crying
Source: Dev Psychobiol. 2020 Nov 17;63(5):1399–414. doi: 10.1002/dev.22058 (PMC8451806; doi:10.1002/dev.22058)
Supplement: Supplementary file 2 — Table S1 [file DEV-63-1399-s001.docx]

**Supplemental Materials Table 1.** Correlations of the observed variables in the non-imputed dataset.

|  |  | **Age** | **Edu** | **EPDS** | **Maltr** | **HG** | **CC** | **Bi cingulum** | **Bi inf FOF LF** | **Bi sup FOF** | **Bi sup LF** | **Bi UF** |
| --- | --- | --- | --- | --- | --- | --- | --- | --- | --- | --- | --- | --- |
| Age | *r* |  | -0.04 | -0.07 | 0.08 | -0.05 | -0.08 | 0.02 | -0.06 | -0.14 | -0.19^*^ | 0.01 |
|  | *N* |  | 120 | 116 | 116 | 118 | 121 | 121 | 121 | 121 | 121 | 121 |
| Edu | *r* |  |  | 0.03 | -0.18 | 0.15 | -0.04 | 0.01 | -0.03 | -0.01 | -0.08 | -0.08 |
|  | *N* |  |  | 116 | 116 | 118 | 120 | 120 | 120 | 120 | 120 | 120 |
| EPDS | *r* |  |  |  | 0.15 | 0.01 | 0.09 | 0.08 | 0.05 | 0.03 | 0.07 | -0.12 |
|  | *N* |  |  |  | 116 | 114 | 116 | 116 | 116 | 116 | 116 | 116 |
| Maltr | *r* |  |  |  |  | 0.22^*^ | 0.04 | 0.00 | 0.11 | -0.04 | -0.09 | 0.01 |
|  | *N* |  |  |  |  | 114 | 116 | 116 | 116 | 116 | 116 | 116 |
| HG | *r* |  |  |  |  |  | -0.06 | 0.01 | -0.01 | -0.07 | 0.08 | 0.00 |
|  | *N* |  |  |  |  |  | 118 | 118 | 118 | 118 | 118 | 118 |
| CC | *r* |  |  |  |  |  |  | 0.64^**^ | 0.57^**^ | 0.48^**^ | 0.57^**^ | 0.43^**^ |
|  | *N* |  |  |  |  |  |  | 121 | 121 | 121 | 121 | 121 |
| Bi cingulum | *r* |  |  |  |  |  |  |  | 0.52^**^ | 0.38^**^ | 0.49^**^ | 0.40^**^ |
|  | *N* |  |  |  |  |  |  |  | 121 | 121 | 121 | 121 |
| Bi inf FOF LF | *r* |  |  |  |  |  |  |  |  | 0.41^**^ | 0.45^**^ | 0.36^**^ |
|  | *N* |  |  |  |  |  |  |  |  | 121 | 121 | 121 |
| Bi sup FOF | *r* |  |  |  |  |  |  |  |  |  | 0.43^**^ | 0.26^**^ |
|  | *N* |  |  |  |  |  |  |  |  |  | 121 | 121 |
| Bi sup LF | *r* |  |  |  |  |  |  |  |  |  |  | 0.38^**^ |
|  | *N* |  |  |  |  |  |  |  |  |  |  | 121 |
| Bi UF | *r* |  |  |  |  |  |  |  |  |  |  |  |
|  | *N* |  |  |  |  |  |  |  |  |  |  |  |

Note. Edu = educational level, EPDS = Edinburgh Postnatal Depression Scale, Maltr = experienced childhood maltreatment, HG = residualized handgrip force ratio, CC = corpus callosum, Bi = bilateral, inf = inferior, FOF = fronto-occipital fasciculus, LF = longitudinal fasciculus, sup = superior UF = uncinate fasciculus, * = p<.05 (2-tailed), ** = p<.01 (2-tailed)
