# Supplementary material for: White matter integrity moderates the relation between experienced childhood maltreatment and fathers’ behavioral response to infant crying
Source: Dev Psychobiol. 2020 Nov 17;63(5):1399–414. doi: 10.1002/dev.22058 (PMC8451806; doi:10.1002/dev.22058)
Supplement: Supplementary file 3 — Table S2 [file DEV-63-1399-s005.docx]

**Supplemental Materials Table 2.** Results for the exploratory moderation analyses in the non-imputed dataset for the UF (*N* = 113), testing direct effects of white matter integrity (i.e., mean skeletonized FA values) on handgrip force in reaction to infant crying, as well as interaction effects with experienced maltreatment scores on handgrip force.

|  | **Β *(SE)*** | ***t*** | ***p*** | ***CI*** |
| --- | --- | --- | --- | --- |
| Constant | 0.00 (0.09) | -0.04 | .97 | [-0.18, 0.17] |
| Age | 0.00 (0.02) | 0.06 | .95 | [-0.04, 0.05] |
| Edu | 0.10 (0.05) | 1.91 | .06 | [0.00, 0.21] |
| EPDS | -0.02 (0.03) | -0.52 | .60 | [-0.07, 0.04] |
| Maltreatment | 0.28 (0.11) | 2.66 | .01 | [0.07, 0.49] |
| Bi UF | -0.54 (2.70) | -0.20 | .84 | [-5.90, 4.82] |
| Bi UF x Maltreatment | -8.38 (3.01) | -2.78 | .01 | [-14.35, -2.41] |

Note. Edu = Educational level, EPDS = Edinburgh Postnatal Depression Scale, Bi = bilateral, UF = uncinate fasciculus
